# Supplementary material for: Unexpected Amanita phalloides-Induced Hematotoxicity—Results from a Retrospective Study
Source: Toxins (Basel). 2024 Jan 29;16(2):67. doi: 10.3390/toxins16020067 (PMC10891511; doi:10.3390/toxins16020067)
Supplement: Supplementary file 1 [file toxins-16-00067-s001.zip › toxins-2807858-supplementary.pdf]

toxins

Supplementary Materials: Unexpected *Amanita phalloides*-Induced Hematotoxicity—Results from a Retrospective Study

Miranda Visser, Willemien F. J. Hof, Astrid M. Broek, Amanda van Hoek, Joyce J. de Jong, Daan J. Touw and Bart G. J. Dekkers

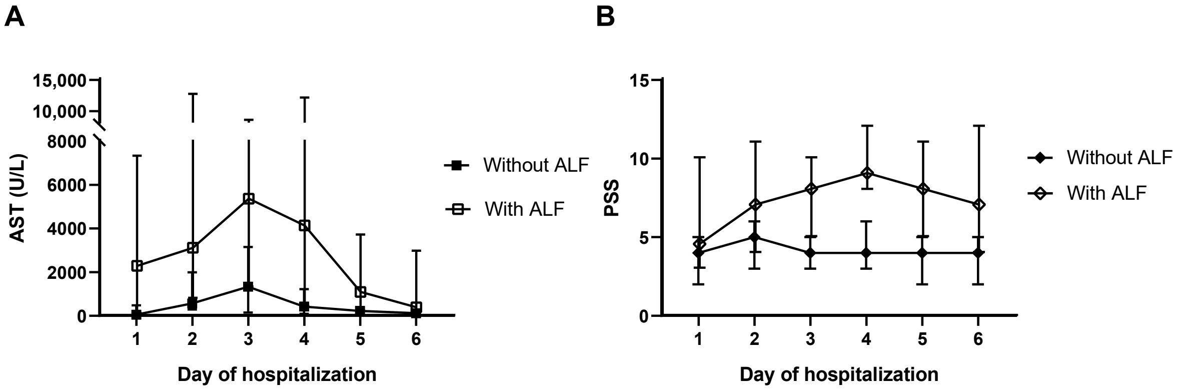

Supplementary Figure S1. *(**A**) AST concentrations and (**B**)* PSS scores *over the time in patients with or without ALF. Data represent medians and range of 22 patients without ALF and 6 patients with ALF.*

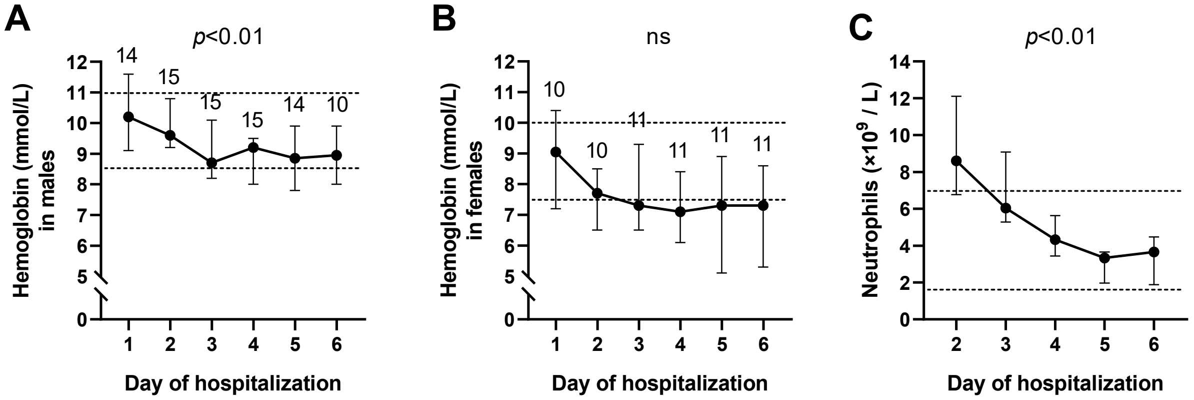

Supplementary Figure S2*. Effect of Amanita phalloides poisoning on hemoglobin concentrations in (**A**) males and (**B**) females. The number of patients is indicated above the data points. (**C**) Neutrophil cell number during hospitalization (n=3). Upper and lower reference values are indicated by the dotted lines. Data represent medians and range of 3 to 15 patients. ns=not statistically significant.*

toxins MDPI

Supplementary Table S1: Blood parameters at day 1-6 after Amanita phalloides *poisonings.*

**Blood cell count parameters**	Day 1	Day 2	Day 3	Day 4	Day 5	Day 6
Hematocrit (*n*=19)	0.470 (0.331 – 0.566)	0.404 (0.301 – 0.537)	0.394 (0.303 – 0.500)	0.377** (0.258 – 0.466)	0.370** (0.180 – 0.494)	0.382** (0.230 – 0.519)
- Males (*n*=11)	0.500 (0.418 – 0.566)	0.454 (0.380 – 0.537)	0.450 (0.346 – 0.500)	0.406* (0.350 – 0.466)	0.399* (0.344 – 0.494)	0.420 (0.327 – 0.519)
- Females (*n*=8)	0.446 (0.331 – 0.530)	0.364 (0.301 – 0.530)	0.334 (0.303 – 0.367)	0.290 (0.258 – 0.378)	0.273 (0.180 – 0.403)	0.345 (0.230 – 0.398)
Mean Corpuscular Volume (MCV) (*n*=16)	88.1 (77.7 – 98.5)	87.5 (66.0 – 99.1)	87.9 (76.0 – 99.8)	86.7 (79.0 – 96.0)	85.3 (65.5 – 95.0)	85.7 (65.8 – 95.3)
Erythrocytes (*n*=4)	5.76 (5.20 – 6.54)	5.45 (4.82 – 6.48)	5.35 (4.68 – 5.86)	5.06 (4.34 – 5.67)	5.15 (4.44 – 5.77)	4.86 (4.77 – 5.66)
Microcytic erythrocytes (*n*=3)	1.5 (1.1 – 3.9)	1.7 (0.9 – 4.2)	1.8 (1.7 – 5.6)	3.2 (1.8 – 7.2)	2.9 (1.2 – 4.2)	2.3 (1.1 – 3.7)
Reticulocytes (*n*=3)		68.8 (55.5 – 104.0)	67.6 (60.4 – 74.8)	43.0 (34.9 – 66.3)	56.8 (36.8 – 78.1)	68.2 (34.5 – 91.7)
Immature reticulocyte fraction (*n*=3)		7.9 (7.7 – 10.7)	10.3 (9.1 – 11.5)	8.1 (7.7 – 14.3)	12.2 (7.5 – 15.8)	15.8 (5.8 – 17.1)
Eosinophils (*n*=3)		0.00 (0.00 – 0.01)	0.01 (0.00 – 0.01)	0.25 (0.22 – 0.26)	0.37* (0.30 – 0.53)	0.43* (0.30 – 0.84)
Immature granulocytes (*n*=3)		0.06 (0.04 – 0.06)	0.03 (0.03 – 0.07)	0.03 (0.02 – 0.06)	0.02 (0.01 – 0.04)	0.03 (0.02 – 0.04)
Lymphocytes (*n*=3)		0.62 (0.58 – 1.88)	1.15 (0.71 – 1.28)	1.63 (1.08 – 1.84)	2.38 (1.21 – 2.93)	2.76 (1.38 – 3.18)

toxins MDPI

Supplementary Table S1 continued: Blood parameters at day 1-6 after Amanita phalloides *poisonings.*

**Blood cell count parameters**	Day 1	Day 2	Day 3	Day 4	Day 5	Day 6
Basophils (*n*=3)		0.02 (0.01 – 0.04)	0.02 (0.01 – 0.03)	0.04 (0.01 – 0.05)	0.04 (0.02 – 0.07)	0.06 (0.03 – 0.07)
Monocytes (*n*=3)		1.02 (0.45 – 1.08)	0.67 (0.39 – 0.75)	0.71 (0.35 – 0.76)	0.64 (0.59 – 0.95)	0.79 (0.78 – 0.91)
Platelet large cell ratio (*n*=3)	26.5 (26.3 – 47.7)	29.8 (25.7 – 45.5)	34.5 (30.0 – 48.8)	33.1 (26.1 – 43.9)	33.9 (27.4 – 49.9)	36.5 (29.9 – 44.4)

Data represent medians and range. * $p < 0.05$, ** $p < 0.01$, *** $p < 0.001$.
